# Predicting potential distribution of the *Rhinoncus sibiricus* under climatic in China using MaxEnt

**DOI:** 10.1371/journal.pone.0297126

**Published:** 2024-01-19

**Authors:** Wanyou Liu, Huanwen Meng, Baozhu Dong, Jinyu Fan, Xiaoqing Zhu, Hongyou Zhou

**Affiliations:** 1 College of Horticulture and Plant Protection, Inner Mongolia Agricultural University, Hohhot, Inner Mongolia, China; 2 Key Laboratory of Biopesticide Creation and Resource Utilization of Inner Mongolia Higher Education Institution, China; 3 Bureau of Agriculture and Herding, Chifeng City, Inner Mongolia, China; 4 Ulangab Institute of Agricultural and Forestry Sciences, Ulangab, Inner Mongolia, China; Baotou Medical College, MONGOLIA

## Abstract

In recent years, buckwheat (*Fagopyrum* spp.) is being increasingly damaged by the Siberian tortoise beetle (*Rhinoncus sibiricus* Faust). Adults and nymphs feed on leaf tissues and caulicles, thus damaging its stems and leaves. In this study, we investigated the habits, distribution, and environmental impact of *R*. *sibiricus* using MaxEnt, an ecological niche model. Geographic information about the infestation site from previous field surveys and climatic data from 2013 to 2018 were organized and optimized using R. The impact factors were calculated using MaxEnt software. The results indicate that population fluctuations in *R*. *sibiricus* are related to changes in temperature, humidity, and their spatial distribution. Under current climatic conditions, *R*. *sibiricus* is mainly distributed in northern China, with sporadic distribution in south–western China. The values for a survival probability threshold > 0.3 were: precipitation during the wettest month (bio13), 70.31–137.56 mm; mean temperature of the coldest quarter (bio11), -15.00–0.85°C; mean temperature of the warmest quarter (bio10), 11.88–23.16°C; precipitation during the coldest quarter (biol9), 0–24.39 mm. The main factors contributing > 70% to the models were precipitation during the wettest month and coldest quarter, and mean temperature during the warmest and coldest quarters. Under both future climate models, the center of the fitness zone moves northward. Our results will be useful in guiding administrative decisions and support farmers interested in establishing control and management strategies for *R*. *sibiricus*. This study could also serve as a reference for future research on other invasive pests.

## 1 Introduction

Buckwheat (*Fagopyrum esculentum*) is rich in resistant starch, minerals, and proteins; in particular, the abundance of phenolic compounds may help reduce the risk of several chronic conditions such as hypertension, obesity, cardiovascular diseases, and gallstone formation [1]. Buckwheat is found in China, the Russian Federation, France, and Ukraine, and its annual yield exceeds 100 000 tons (1×10^8^ kg) [2].

Buckwheat crops are being destroyed by the Siberian tortoise weevil, *Rhinoncus sibiricus* Faust (Coleoptera: Curculionidae), a rapidly spreading insect pest. This beetle is dark grey in color, with an average body length of 2.5–3.5 mm, and a marking on the body in front of the top suture. It’s rostrum reaches the mid-chest, and the upper part of the rostrum is attached to club-shaped cranked feelers. The males have 8-segmented feelers, while males have 7-segmented feelers. These beetles have large forward-directed eyes, well-developed mouths, and hard and oval-shaped upper wings. The front and back portions of the notum are in the same horizontal plane [3].

*R*. *sibiricus* was first reported to damage buckwheat in eastern Russia by Mishchenko Safiullina in 1952–1957, before being observed in Korea, Japan, Mongolia, and China, with stem damage rates of up to 70% [4]. In China, it was first reported to damage buckwheat in Chifeng, a city in the northeast of China in 2013, and posed a serious threat to buckwheat cultivation [5]. Meng et al. showed that *R*. *sibiricus* was widely distributed across buckwheat planting regions in China, including inner Mongolia and the Ningxia, Hebei, Jilin, and Gansu provinces. It was more prevalent in northern China than in southern China. In addition, the stem damage rate was lower in southern buckwheat-producing areas than northern ones [6].

*R*. *sibiricus* is capable of causing significant damage throughout the entire buckwheat growing season. The adult beetles appear during the end of May and start of June, feed on buckwheat seedlings (which grow from seeds that were accidentally sown the previous year, including those of the smartweed-buckwheat *Polygonaceae* Juss). Adult beetles consume the seedling leaves and created holes, leading to irregular leaf edges. The larvae burrow in the stems and nodes of the plants and disrupt the vascular conducting system, which causes increased lodging of the plants, reduced nectar secretion, and decreased crop yields [7].

In some years, the beetles may damage 100% of the leaves, leading to shoot death. The stem damage in the maturity stage varied from 67,7% till 92,5%. The buckwheat cultivation area in Russia decreased from 30 000 hm^2^ in 1990–1995 to 3000 hm^2^ in 2011 because of *R*. *sibiricus* infestations [8]. The *R*. *sibiricus* spread to Chifeng in Inner Mongolia, China, and destroyed 3666.67 hm^2^ of buckwheat farmland in 2013 [9]. Our surveys of *R*. *sibiricus* infestations from 2013–2018 revealed that this pest has seriously affected buckwheat crop production throughout China. *R*. *sibiricus* is a rapidly emerging pest in China, and has dispersed rapidly across buckwheat-producing regions. Existing studies on *R*. *sibiricus* have focused on chemical control of this species [10], simple morphological studies [11], its feeding preferences, and damage distribution [6]. Little research has been done on its current distribution, its habitat patterns and dynamics in relation to climate change, and the environmental factors necessary for widespread infestation. Therefore, it is necessary to investigate the ecological habits of *R*. *sibiricus* and predict its distribution in the main buckwheat-producing areas in China, in order to develop novel control strategies.

MaxEnt is an ecological niche model used to predict the distribution of wildlife habitats [12]. It is reliable, direct, and can easily acquire data [13]. It can also predict the distribution of habitats suitable for insect pests. We predicted the climates and regions conducive for *R*. *sibiricus* proliferation in China. Furthermore, the incidence and severity of *R*. *sibiricus* infestations in China could also be forecast from the research data. Based on these findings, we speculated the vulnerability of *R*. *sibiricus* during overwintering and egg-laying periods, which indirectly affect the level of damage that this pest may cause. The aim of this research was to determine the habits of *R*. *sibiricus* and the main environmental factors affecting its growth and development. This study provides a good theoretical basis for developing programs to control *R*. *sibiricus* and ensure the safety of buckwheat production.

## 2 Materials and methods

### 2.1 Data on species occurrence

To identify the most suitable habitats for *R*. *sibiricus* and the effects of climate change on its distribution, field sampling was performed in major buckwheat-producing areas prior to the annual buckwheat harvest during the 2013–2018 period. The geographic coordinates of *R*. *sibiricus* were recorded using a handheld GPS unit (Beijing Yujie Beidou Technology Co., Ltd., Beijing, China). Data from several sampling sites, such as Taiwan, were acquired from the Global Biodiversity Information Facility (GBIF: http://www.gbif.org/). Over the 6-year study period, 148 data points were collected and collated (S1 Table).

**Fig 1:**
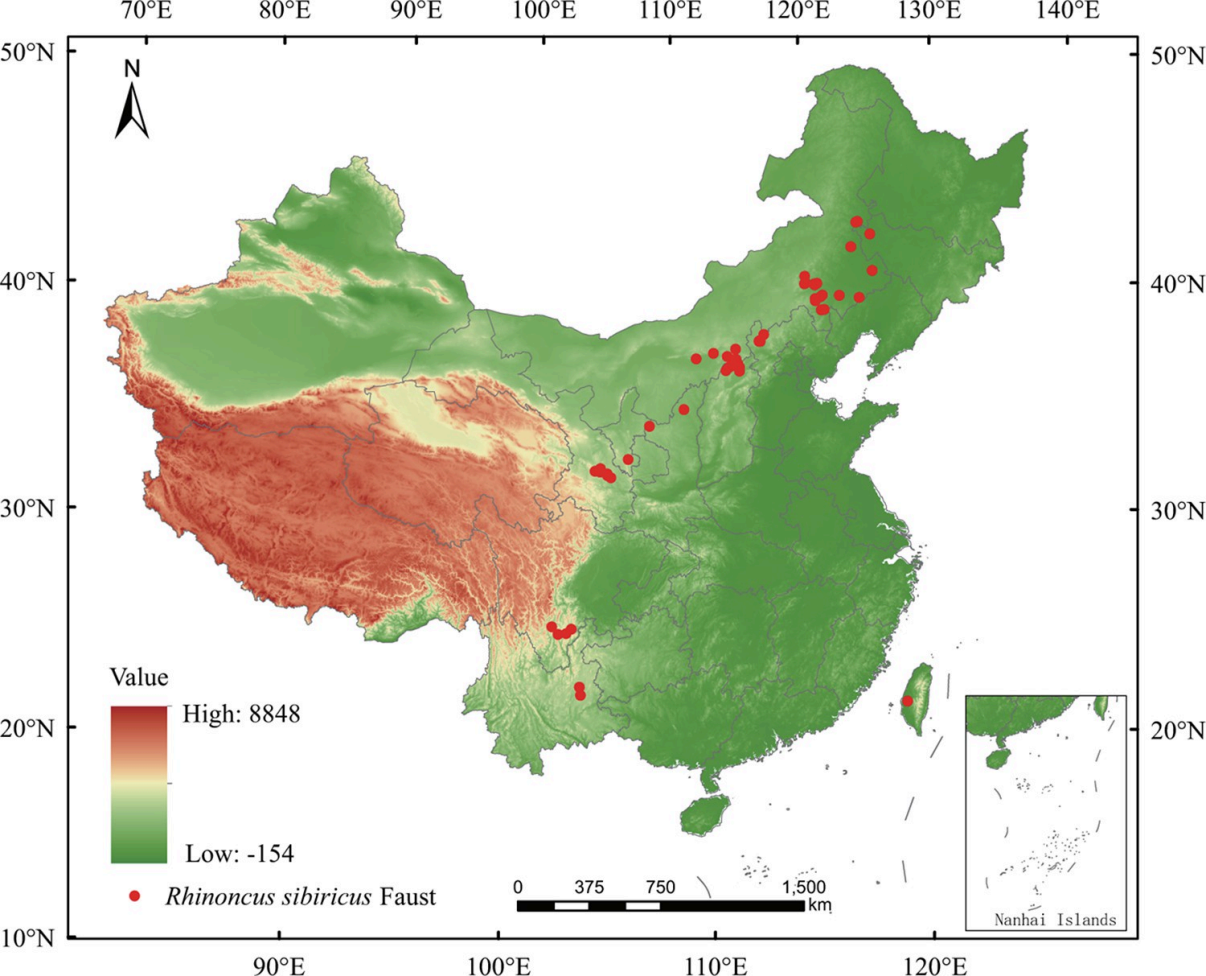
Study area and distribution of *Rhinoncus sibiricus* (Siberian tortoise weevils). General distribution of *R*. *sibiricus* in China. Red dots on the map represent filtered incidences of *R*. *sibiricus*. The boundary was obtained from Natural Earth (http://www.naturalearthdata.com/). Based on the principles of national and territorial integrity, we modified and adjusted the vector boundary.

Occurrence data were filtered in two steps to ensure that they were of acceptable quality. Data were carefully screened for locations lying outside the known range of the species. Records with uncertain or missing coordinates were removed. To reduce autocorrelation between the occurrence data and overfitting potential, one of each pair of records within single-grid cells (~1 km) was eliminated using the spThin library in R v. 3.5.1 [14]. The final filtered distribution data are shown (Fig 1).

### 2.2 Environmental data

Climate, soil, and topography were selected as environmental factors for the study. The climatic factors refer primarily to the bioclimatic variables provided by WorldClim v. 2.0 (http://www.worldclim.org) and represent the main habitat conditions of the species [15]. The spatial resolution was 30" (~1 km). These climatic conditions directly or indirectly affect basic physiological processes, such as growth and development. The 19 bioclimatic variables of WorldClim have been abbreviated in this paper as bio1–bio19. For the climate change projections, the climate variables under future climate scenarios were downloaded to match the current climate change dataset. The latter also has a spatial resolution of 30". The intervals, 2021–2040, 2041–2060, 2061–2080, and 2081–2100, were selected to generate lower and higher estimates of potential future greenhouse gas emissions under two representative concentration path scenarios, namely ssp126 and ssp585.

*Rhinoncus sibiricus* overwinters in the soil in the adult form [4]. Moreover, soil affects buckwheat distribution and, consequently, *R*. *sibiricus* distribution. Hence, the soil factor was also considered, and relevant data were obtained from the World Soil Database (HWSD; http://www.fao.org/soils-portal/soil-survey/), which were consistent with the climate factor data.

The topographic data (spatial resolution of 30") primarily consisted of the digital elevation model (DEM), slope, and slope direction. These factors reflect the differences in the topography of the areas where the insects survive. The DEM data were obtained from the original ASTER data using the Mosaic to NewRaster tool in ArcGIS v 10.6.1 (ESRI, Redlands, CA, USA; https://www.arcgis.com/index.html). Slope and aspect data were processed using the DEM with the Slope and Aspect tools in ArcGIS, respectively.

Multicollinearity among the environmental variables (bioclimatic, soil, and topographic) was tested, and the Composite Bands tool in ArcGIS was used to fuse the three environmental variables into a single layer [16]. Pearson’s correlation coefficients were then computed for each layer using the Band Collection Statistics tool in ArcGIS. Thereafter, the environmental variable matrix was correlated (S1 and S2 Figs). The topographic variables were discontinuous and not directly included in the model. The Pearson’s correlation coefficient (R) between variables was > 0.75 [17]. Ease of use of the environmental variables and their potential ecological relevance to *R*. *sibiricus* were considered [18]. Pearson’s correlation coefficients among variables across the calibration area were calculated using R v. 3.5.0 [19].

Finally, 24 factors were obtained by proposing some of the 57 factors in the three categories of climate, soil, and topography according to the Pearson correlation coefficient (R) > 0.75 among the variables; these factors were subsequently included in the model construction. Finally, 10 factors with high contributions were selected according to the factor contribution rate output using MaxEnt optimization.

### 2.3 Model simulation, optimization, and evaluation

The species distribution models were developed using MaxEnt v. 3.3 [20, 21], a tool widely used for this purpose [12]. Its predictive performance is consistently high [14] and it has successfully produced models from small datasets [22, 23], such as those for rare or elusive species [14]. These models have also been used to study invasive species despite the challenges inherent to such analyses [24].

We used the Kuenm package (https://github.com/marlonecobos/kuenm, accessed on November 24, 2023) to optimize the regularization multiplier and feature class parameters in the R version 3.6.3 software (Vienna, Austria, https://www.r-project.org/, accessed on November 24, 2023) [20]. These two parameters are essential for building the species distribution model with the MaxEnt version 3.4.4 software (New York, NY, USA, https://biodiversityinformatics.amnh.org/open_source/maxent/, accessed on November 24, 2023). In the modeling, 75% of the data were used as the training set. A total of 1160 candidate models, with parameters reflecting all combinations of 40 regularization multiplier settings (from 0.1 to 4, interval of 0.1), and 29 feature class combinations were evaluated.

Model selection was based on statistical significance (partial receiver operating characteristic (ROC)), predictive ability (low omission rates), and complexity (AICc values), in that order of priority. First, candidate models were screened to retain those that were statistically significant; second, the set of models were reduced with the omission rate criterion (i.e., <5% when possible); finally, models with the lowest delta AICc values (<2) were selected among the significant and low-omission candidate models [25].

The parameters set by MaxEnt model were “Create response curves,” “Do jackknife to measure variable importance,” “Random seed,” “Write plot data,” “Write background predictions,” “Replicated run type crossvalidate,” “Replicates 10” [26], and “Output format logistic.” In addition, 75% of the distribution data were set as training data, and the remaining 25% were testing data. For *R*. *sibiricus*, each pixel on the possible distribution map was categorized and assigned a number between 0 and 1. These pixels were then classified as habitats of “high” (> 0.5), “moderate” (0.3–0.5), or “low” (0.1–0.3) suitability or “not potential” (< 0.1) habitats.

**Fig 2:**
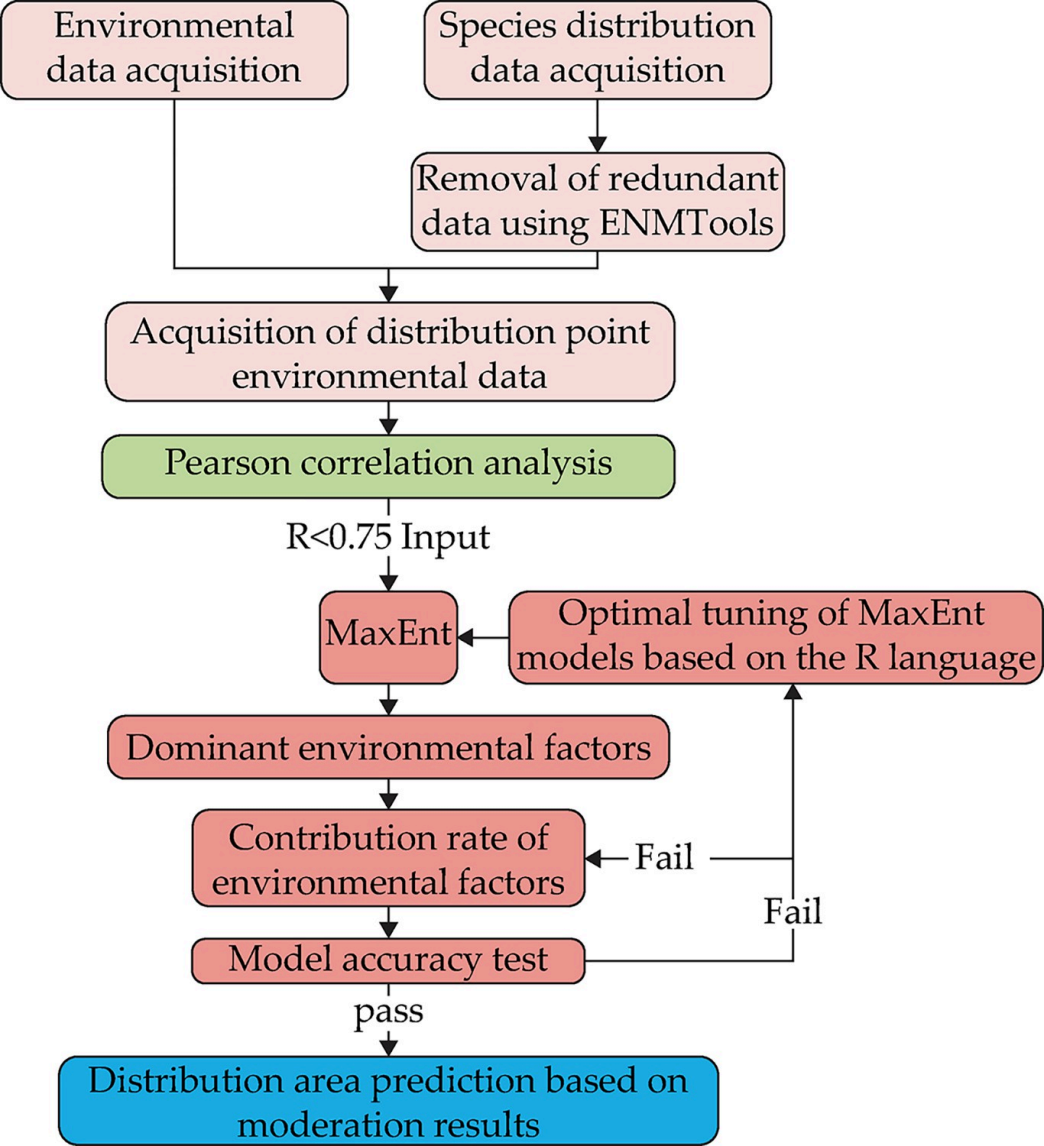
Flowchart of datasets and processes used.

In this study, two widely used model evaluation metrics, area under the curve (AUC) and the true skill statistic (TSS) values were used to evaluate model performance [27]. The AUC value was the area enclosed by the ROC curve and the horizontal coordinate, which was calculated by MaxEnt software and well evaluate the accuracy of the model prediction [28, 29]. The value of AUC ranges from 0 to 1, with a larger the value indicating a higher simulation reliability. The AUC value is divided into five levels: (1) Excellent: 0.90–1.00; (2) Good: 0.80–0.90; (3) General: 0.70–0.80; (4) Difference: 0.60–0.70; (5) Failure: 0.50–0.60 [30]. The data predicted by MaxEnt model and validation data set were used to calculate TSS [31]. The TSS value is based on the maximum specificity and sensitivity thresholds, and the TSS index is calculated as: TSS = sensitivity specificity-1, with TSS values ranging from -1 to 1 [28], where 1 represents the ability to perfectly distinguish between suitable and unsuitable areas. When TSS values are >0.75, the model performs very well [32]. A value of ≤0 indicates that the prediction performance is no better than random prediction [33]. The results of this test are shown in S1 and S2 Files. The combination of AUC value and TSS value can better evaluate the performance of the model [34]. The overall process is shown in Fig 2.

### 2.4 Shifts in the suitable habitat distribution center

The species distribution model (SDM) toolbox (http://www.sdmtoolbox.org/downloads) and the GIS toolkit (gistoolkit.sourceforge.net), which is based on Python (https://www.python.org/downloads/), were used to calculate and compare centroids between present and future regions with habitats suitable for *R*. *sibiricus*. The SDM toolbox (including present and future SDMs) was used to calculate changes in distribution [35]. Species distributions were then reduced to a single centroid point. Magnitudes and directions were created for estimated time-dependent variations. Movements of the centroids of various SDMs were tracked to examine the shifts in distribution.

## 3 Results

### 3.1 Species distribution model and its accuracy

**Fig 3:**
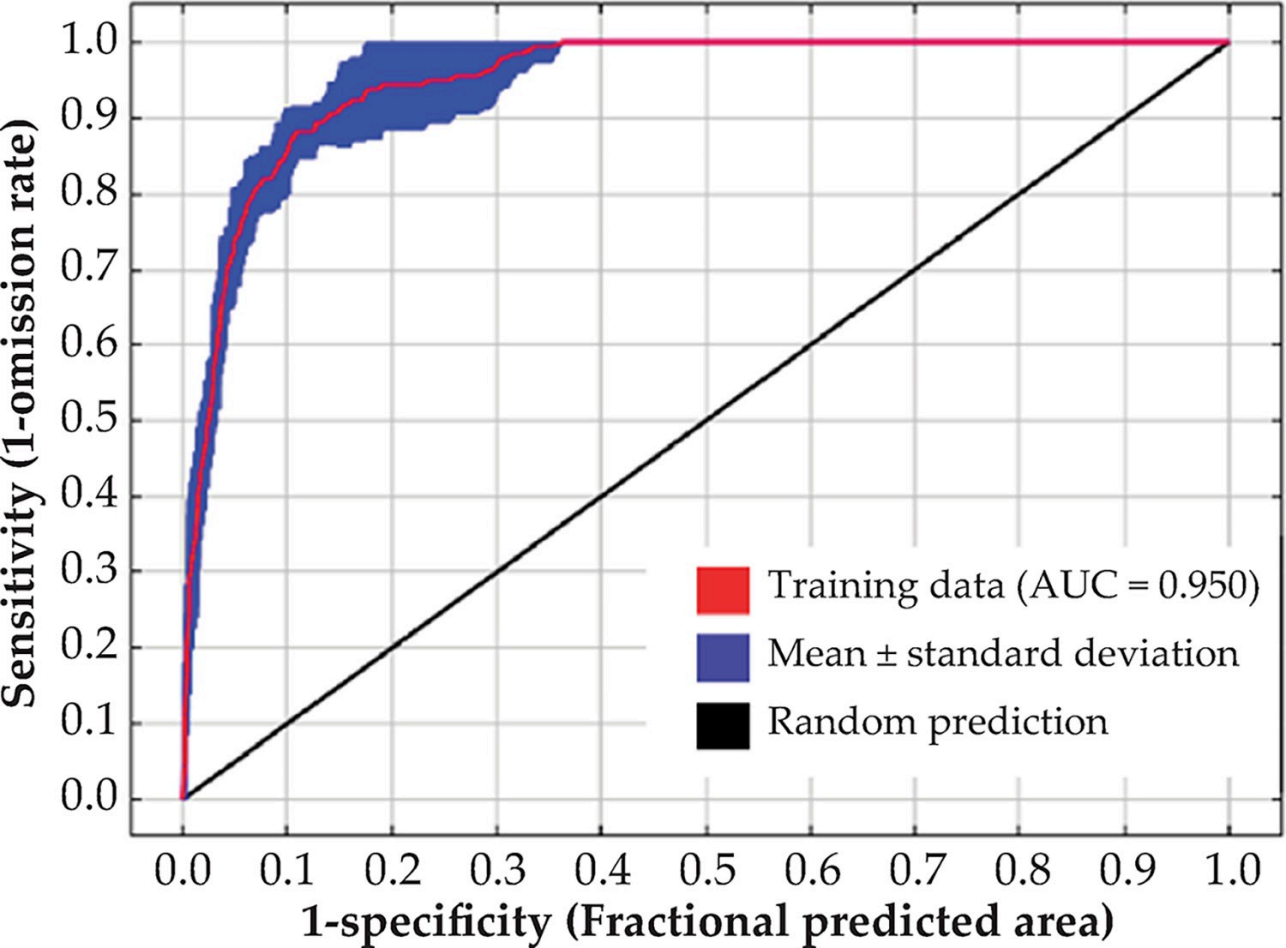
Receiver operating characteristic (ROC) curve for evaluating the distribution of *R*. *sibiricus* based on the MaxEnt model. Area under the curve (AUC) was used to assess model prediction effectiveness. The model predicted *R*. *sibiricus* distribution with high confidence (AUC for training data = 0.950).

**Fig 4:**
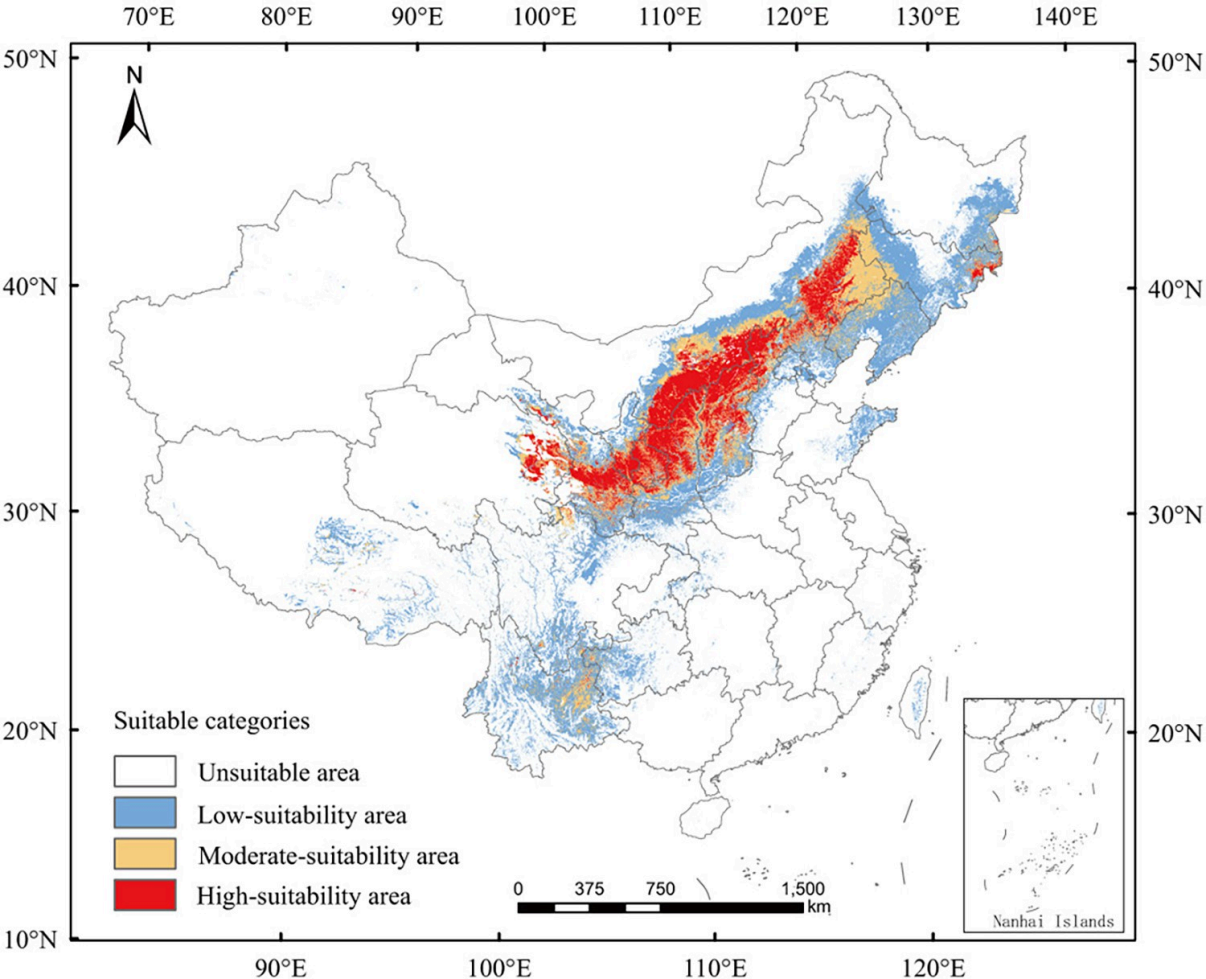
Predicted current suitable regions for *Rhinoncus sibiricus* distribution in China. Area distribution of suitable *R*. *sibiricus* habitats under current climatic conditions. Red: high-suitability area, yellow: moderate-suitability area, blue: low-suitability area, white: unsuitable area. The boundary was obtained from Natural Earth (http://www.naturalearthdata.com/). Based on the principles of national and territorial integrity, we modified and adjusted the vector boundary.

For the test set, the model predicted AUC and TSS values of 0.950 and 0.785, respectively. Hence, the model performance was acceptable (Fig 3 and S3 Fig). The current total area of the *R*. *sibiricus* habitats that were moderately and highly suitable was 109.364 × 105 km^2^. These areas were mainly distributed in eastern and central Inner Mongolia, Shaanxi, northern Shanxi, southeastern Gansu, southern Ningxia, eastern Qinghai, and parts of Sichuan and Yunnan (Fig 4).

### 3.2 Important environmental parameters affecting habitat distribution of *R*. *sibiricus*

**Fig 5:**
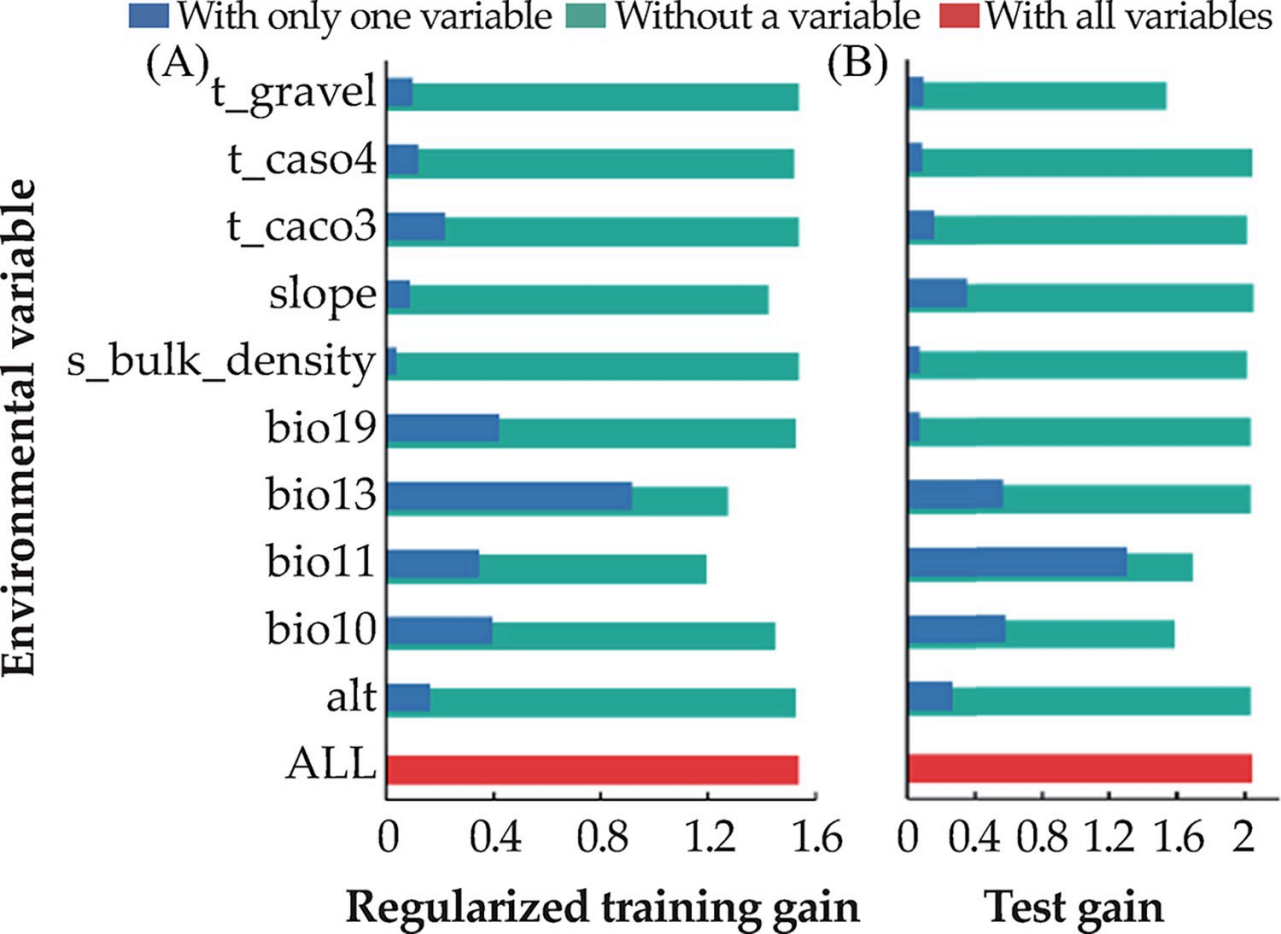
Jackknife test for determining the effect of major variables on *Rhinoncus sibiricus* distribution. A: Regularized training gain for *R*. *sibiricus* (full model); B: Test gain for *R*. *sibiricus*. Effects of variables in the sample set were used to validate the model.

The jackknife test of variable importance revealed that among the environmental variables, precipitation during the wettest month (bio13) was associated with the highest gain. This factor contained the most useful information. The mean temperature of the coldest quarter (bio11) was the environmental variable that reduced the gain to the maximum extent when omitted. Hence, it was associated with the most information absent in the other variables (Fig 5).

The internal jackknife test of factor importance revealed that, when compared to the other variables, precipitation during the wettest month (bio13; 27.5% contribution), precipitation during the coldest quarter (bio19; 17.5% contribution), mean temperature of the coldest quarter (bio11; 16.5% contribution), meters above sea level (alt; 10.6% contribution), and mean temperature of the warmest quarter (bio10; 10.3% contribution) contributed the most to the *R*. *sibiricus* distribution model. The cumulative contribution of these factors was as high as 82.4% (Table 1).

**Table 1:** Environmental variables considered in the *Rhinoncus sibiricus* MaxEnt model and their contributions.

Variable	Environment variable	Unit	Contribution percentage	Permutation importance
bio13	Precipitation during the wettest month	mm	27.5	37.8
bio19	Precipitation during the coldest quarter	mm	17.5	1
bio11	Mean temperature of the coldest quarter	°C	16.5	26.7
alt	Meters above sea level	m	10.6	4.9
bio10	Mean temperature of the warmest quarter	°C	10.3	21.6
slope	-		8.9	5.6
t_caso4	Sulfate content		4.3	1.7
t_caco3	Carbonate or lime content		3.7	0.2
s_bulk_density	Surface soil bulk density		0.7	0.3
t_gravel	Percentage volume of gravel	%	0.1	0.2

**Fig 6:**
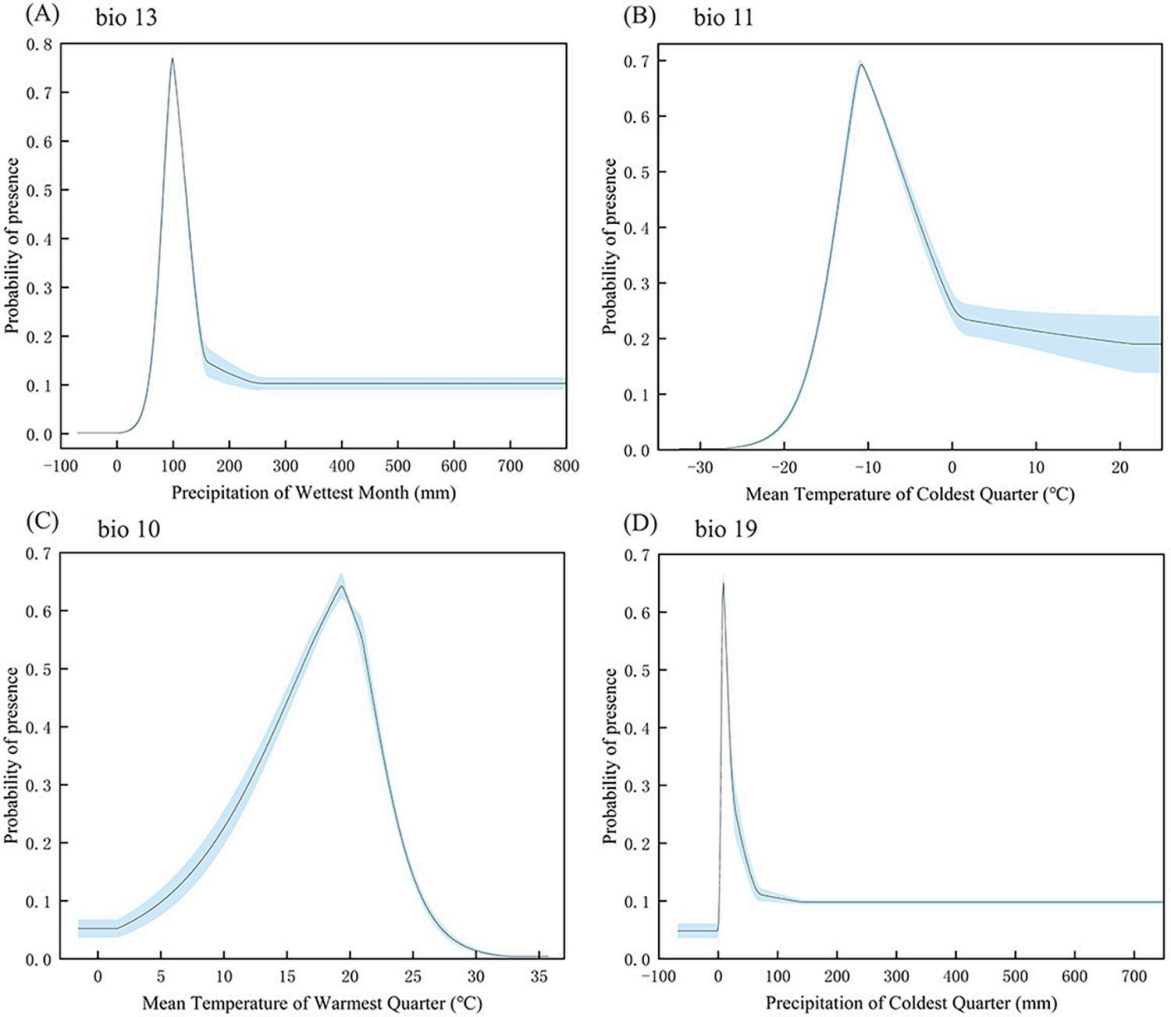
Response curves of important environmental indices in the *Rhinoncus sibiricus* distribution model. Response Curves for (A): precipitation during the wettest month; (B): mean temperature of the coldest quarter; (C): Response curve for mean temperature of the warmest quarter; (D): precipitation during the coldest quarter.

Four important variables were selected to plot the response curves from the combined results of the jackknife test, internal jackknife test, and ecological significance. In MaxEnt, the probability threshold for existence was > 0.3 for the main bioclimatic parameters. Their values were in the following range: precipitation during the wettest month (bio13), 70.31–137.56 mm; mean temperature of the coldest quarter (bio11), -15.00°C to -0.85°C; mean temperature of the warmest quarter (bio10), 11.88°C to 23.16°C; precipitation during the coldest quarter (bio19), 0–24.39 mm. Hence, *R*. *sibiricus* thrived under warm dry winters and cool dry summers (Fig 6).

### 3.3 Variations in the future spatial extent of suitable habitats

**Fig 7:**
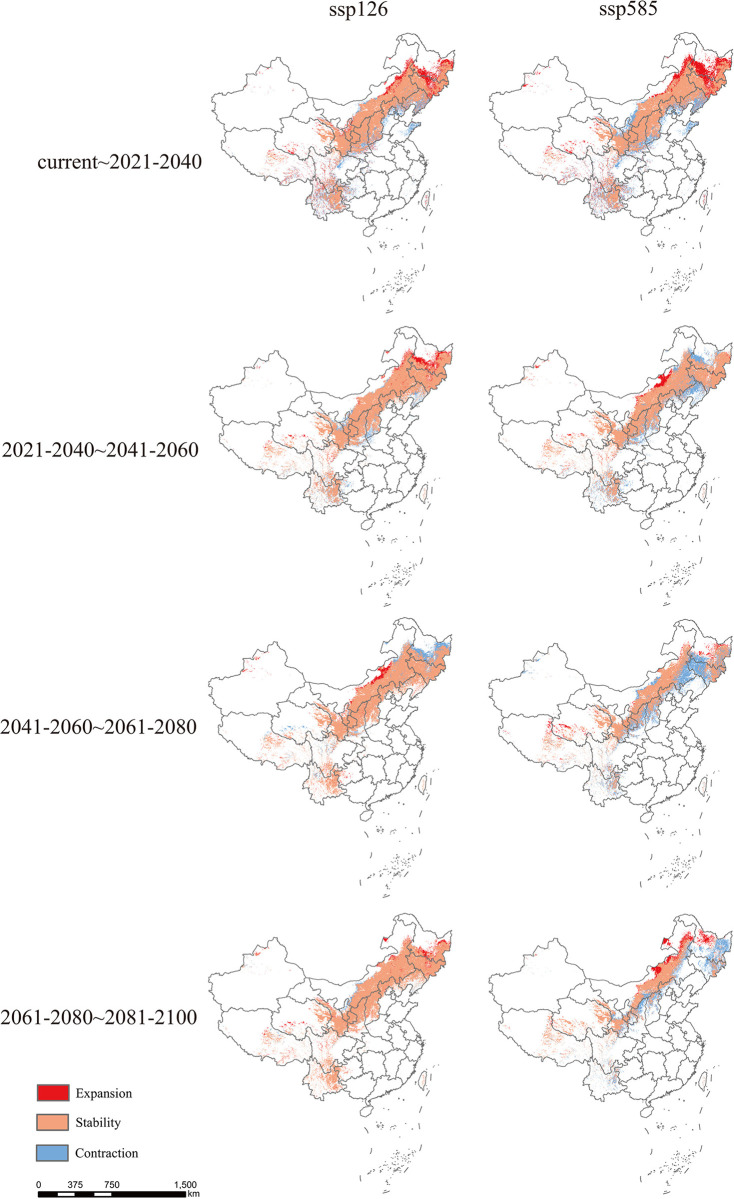
Prediction of suitable *Rhinoncus sibiricus* distributions using MaxEnt under climate models ssp126 and ssp585. Climate models are based on low and high carbon emissions. Regional changes in *R*. *sibiricus* distribution in China were predicted for the current period (2021–2040), 2021–2040 to 2041–2060, 2041–2060 to 2061–2080, and 2061–2080 to 2081–2100 (red: newly expanded areas, orange: stable areas, and blue: contracted areas). The boundary was obtained from Natural Earth (http://www.naturalearthdata.com/). Based on the principles of national and territorial integrity, we modified and adjusted the vector boundary.

Based on the ssp126 and ssp585 scenarios, we predicted where the area of occurrence of suitable habitats might be concentrated. Large patches appeared in east-central Inner Mongolia, most of Liaoning, all of Jilin, Northern Hebei, Southern Heilongjiang, Shanxi, most of Shaanxi, and southern Gansu. Smaller scattered patches appeared in Qinghai, Xinjiang, Xizang, Guizhou, and Sichuan (Fig 7). The expansion area decreased in the four periods combined, whereas the area of stable regions remained relatively consistent. The area of contracting regions decreased, except during 2021–2040. The area of expanding regions was mainly located in northeastern Inner Mongolia and central Heilongjiang, whereas the contracting areas were concentrated mainly in southwestern Shanxi. The south–central part of Heilongjiang is projected to expand during 2021–2040 and 2041–2060 but is expected to subsequently contract during 2061–2080 and 2081–2100. The area of occurrence decreased significantly with time in the ssp585 scenario. Most of the expanding areas appear in northeastern Inner Mongolia (Table 2, Fig 7).

**Table 2:** Relative changes in *Rhinoncus sibiricus* habitat area under climate scenarios ssp126 and ssp585.

Area comparison between periods (1 × 10^5^ km^2^)					
		Current (2021–2040)	(2021–2040) to (2041–2060)	(2041–2060) to (2061–2080)	(2061–2080) to (2081–2100)
ssp126	Expansion area	26.788	11.418	8.266	10.016
	Non-habitable area	754.000	770.597	778.080	785.081
	Stability area	146.469	162.237	156.637	161.013
	Contraction area	32.743	15.748	17.018	3.890
ssp585	Expansion area	32.895	7.475	6.069	12.839
	Non-habitable area	747.893	776.207	804.746	842.129
	Stability area	138.639	141.710	98.964	69.920
	Contraction area	40.573	34.608	50.222	35.112

The area of stability for *R*. *sibiricus* was predicted using the MaxEnt model and the ssp585 scenario. The stable area was predicted to decrease to 69.920 × 105 km^2^ by the 2100s; the area was expected to be concentrated mainly in central–eastern Inner Mongolia, southern Gansu, and southern Ningxia, but scattered in Heilongjiang, Jilin, and Qinghai-Xizang (Table 2, Fig 7).

### 3.4 Core distributional shifts

**Fig 8:**
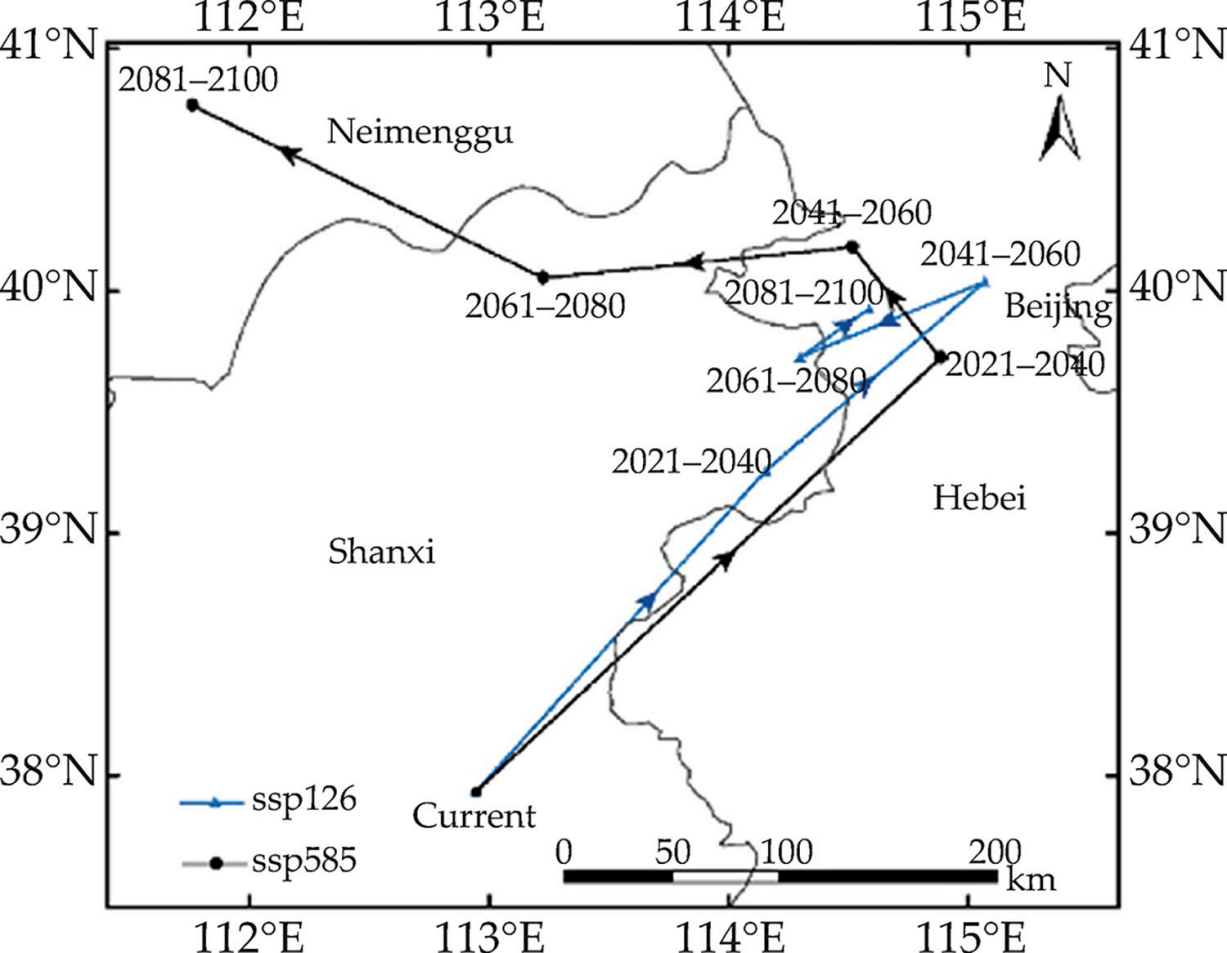
Core distributional shifts under different climate scenarios for *Rhinoncus sibiricus*. Arrows indicate the magnitude and direction of predicted shifts over time. The boundary was obtained from Natural Earth (http://www.naturalearthdata.com/). Based on the principles of national and territorial integrity, we modified and adjusted the vector boundary.

The shift in the center of mass concentration was predicted using the low carbon emission scenario. The centroid of the current *R*. *sibiricus* habitat was located at 112° 56′ E and 37° 58′ N in Shanxi Province (Fig 8). The centroid of the suitable area is expected to shift to 114° 11′ E and 39° 16′ N in the northeast during 2021–2040. During 2041–2060, the suitable core is expected to continue to shift northeastward to 115° 7′ E and 40° 3′ N in Hebei. However, during 2061–2080, the core is expected to slightly shift back to northeastern Shanxi (114° 21′ E and 39° 44′ N). Additionally, during 2081–2100, the core is expected to shift back to the Hebei Province (114° 38′ E and 39° 56′ N).

When the high carbon emission scenario was used, the overall core distribution shifted toward the northeast and then toward the northwest over a larger distance during 2021–2040. The first shift was from the current point in Hebei Province (114° 55′ E and 39° 45′ N) to the south-central region of Inner Mongolia (111° 51′ E and 40° 40′ N).

Over time, the predicted distributions under both carbon emission scenarios shifted toward the higher latitudes. The predicted distributions under the high carbon emission scenario shifted toward the northwest, whereas those under the low carbon emission scenario shifted toward the northeast.

## 4 Discussion

The results of this study indicated that *R*. *sibiricus* was located mainly at the junction of the second- and third-gradient terrains (Fig 4). The main habitat of *R*. *sibiricus* largely overlaps with arid steppe distribution in China [36]. This is also consistent with previous findings, which indicated that the maximum damage to buckwheat crops by *R*. *sibiricus* primarily occurred in the steppe zone [7]. Previous surveys in China have reported the distribution of *R*. *sibiricus* to a large extent in the north and to a far less extent in the south. This study also revealed that *R*. *sibiricus* prefers the dry steppe climate of northern China. These distribution results are consistent with those reported by Meng *et al* [6].

The data (Table 1 and Fig 5) were combined, and all variables were ordered from the largest to the smallest. Only the first four variables were considered for each evaluation method. The analysis showed that the most impactful factors were precipitation during the wettest month (bio13), mean temperature of the coldest quarter (bio11), precipitation during the coldest quarter (bio19), and mean temperature of the warmest quarter (bio10). The overall conclusion is that humidity and temperature have a huge impact on insects, which is consistent with the suggestion that temperature and humidity directly or indirectly affect insect activity, dispersal, and survival, as presented by Jaworski and Hilszczański [37].

The four significant environmental factors can be divided into two parts i.e. summer and winter. Each part contains two critical factors of humidity and temperature. Previous studies indicated that summer and winter corresponded to the oviposition and adult overwintering stages of *R*. *sibiricus*, respectively [5, 7]. Therefore, the overwintering and oviposition periods of *R*. *sibiricus* are sensitive to environmental conditions. Humidity and temperature are vital for it at both two stages of *R*. *sibiricus*

In the summer *R*. *sibiricus* prefers arid climates. This is similar to studies on the bollworm (*Helicoverpa armigera* Huber), which showed that the intensity of rainfall between June and August determined the severity of infestation by this insect pest [38]. According to Fitt [39], insect eggs are sensitive to humidity. We observed that *R*. *sibiricus* thrived during the warmest quarter (bio10) with a mean temperature of 11.88–23.16°C. Therefore, we hypothesize that the eggs of *R*. *sibiricus* may be better suitable for relatively cool summers. Relative humidity and temperature affect egg survival and development duration in *Hypera postica*, which also belongs to the family Curculionidae [22], but the specific quantitative effects for *R*. *sibiricus* may differ from those of *Hypera postica* because of species related differences. Quantitative effects of relative humidity and temperature on egg development in *R*. *sibiricus* should be evaluated in the future studies.

Notably, *R*. *sibiricus* is an overwintering adult and is sensitive to windy and cold weather [4]. This coincides with our prediction that the suitable survival temperature for *R*. *sibiricus* in winter is -15.00°C to -0.85°C. The MaxEnt model used here could effectively predict the patterns of *R*. *sibiricus* occurrence and dissemination. Based on these conclusions, the probability of *R*. *sibiricus* occurrence increases if the main buckwheat production area is dry and cool in summer and dry and warm in winter. According to a previous study, *R*. *sibiricus* primarily feeds on wild buckwheat. In the 1940s, *R*. *sibiricus* was found to be a threat to buckwheat production in the Far East of the Soviet Union [8]. The results of the present study and the GBIF database revealed that *R*. *sibiricus* might be ubiquitous in China, Mongolia, Japan, North Korea, and Palestine. It was detected in these regions after its diet shifted from wild to cultivated buckwheat. The MaxEnt model predicted that *R*. *sibiricus* will continue to threaten buckwheat production well into the next century. The core of the distribution of future *R*. *sibiricus* under both climate models shows a northward shift. Jaworski and Hilszczański [37] showed that changes in insect distribution ranges are influenced by climate change, which may also lead to the adaptation of insects to new host plants. This especially occurs when close relatives of the host plant are present in the new range of phytophagous insects. Rising temperatures are causing species to spread to higher elevations and latitudes, and all the above conclusions are consistent with those derived from our model predictions.

Studies on the worldwide distribution of *R*. *sibiricus* are limited because surveys have focused on the occurrence of *R*. *sibiricus* within China and not elsewhere in the world. In the future, an optimized model derived from this study could be extended to predict the global distribution of *R*. *sibiricus* (within the limits of computing power). In addition, there may be some shortcomings in using only one model, MaxEnt, in this study. To increase reliability, multiple prediction models should be used in the future for prediction and comparison.

## 5 Conclusions

To the best of our knowledge, this is the first study to predict *Rhinoncus sibiricus* distribution and occurrence trends over time under two climate models (high and low carbon emissions) based on actual survey data. Under the current climate, *R*. *sibiricus* is common in China’s major buckwheat-producing areas. It is widely distributed in the north and sporadically distributed in the southwest. The *R*. *sibiricus* prefers the arid steppe climate of northern China. It is vulnerable during overwintering and spawning periods, as it is sensitive to environmental conditions in these stages. The climate models ssp126 and ssp585 predicted that *R*. *sibiricus* would continue to infest and damage buckwheat crops in northern China over the next century. Over time, the fitness zones predicted under both carbon emission scenarios will shift to higher latitudes. Therefore, effective control measures against *R*. *sibiricus* should be developed and implemented according to the distribution and displacement of suitable habitats for this insect pest. This can contribute to production security in major buckwheat-producing regions of the world. Further, this study provides a reference for agricultural producers to use this model in the development of environmentally friendly pest prevention and control programs.

## Supporting information

S1 Fig Analysis of collinearity among 19 climate variables. Collinearity matrix of candidate *R*. *sibiricus* predictor climate variables. Collinearity threshold is R > 0.75. Collinearity of variables increases with the depth of blue and red colors. Correlation strength increases with circle size.
(TIF)

S2 Fig Analysis of collinearity among soil variables. Collinearity matrix of candidate *R*. *sibiricus* predictor soil variables. Collinearity threshold is R > 0.75. Collinearity of variables increases with the depth of blue and red colors. Correlation strength increases with circle size.
(TIF)

S3 Fig TSS values for the *Rhinoncus sibiricus* Faust prediction model. (TIF)

S1 File Test value. (TXT)

S2 File The R package used. (TXT)

S1 Table *Rhinoncus sibiricus* Faust hazard site distribution data. (XLS)
