# Supplementary figures and images for: Predicting potential distribution of the Rhinoncus sibiricus under climatic in China using MaxEnt
Source: PLoS One. 2024 Jan 19;19(1):e0297126. doi: 10.1371/journal.pone.0297126 (PMC10798473; doi:10.1371/journal.pone.0297126)

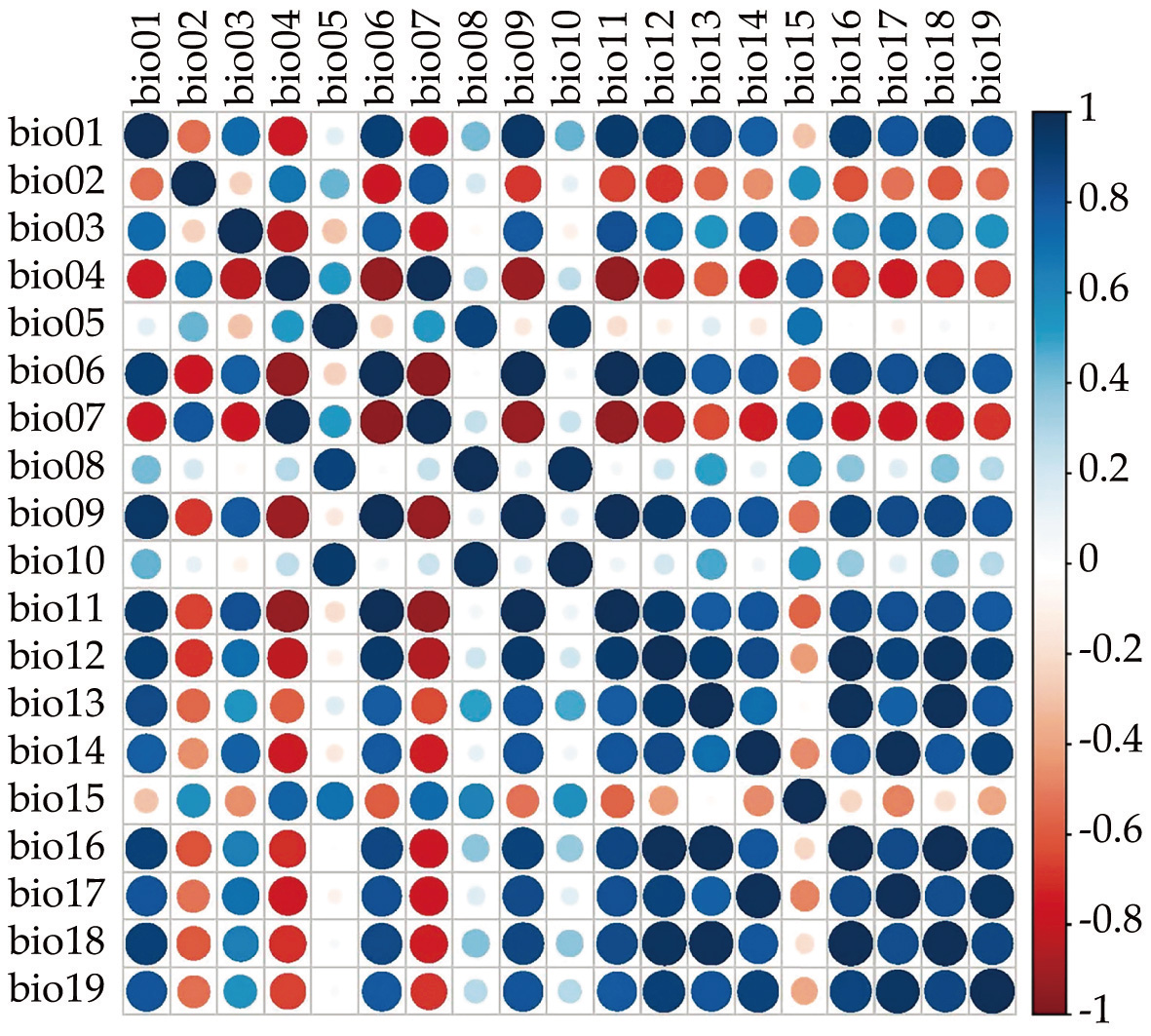

Supplement: S1 Fig — Collinearity matrix of candidate R. sibiricus predictor climate variables. Collinearity threshold is R > 0.75. Collinearity of variables increases with the depth of blue and red colors. Correlation strength increases with circle size. (TIF) [file pone.0297126.s001.tif]

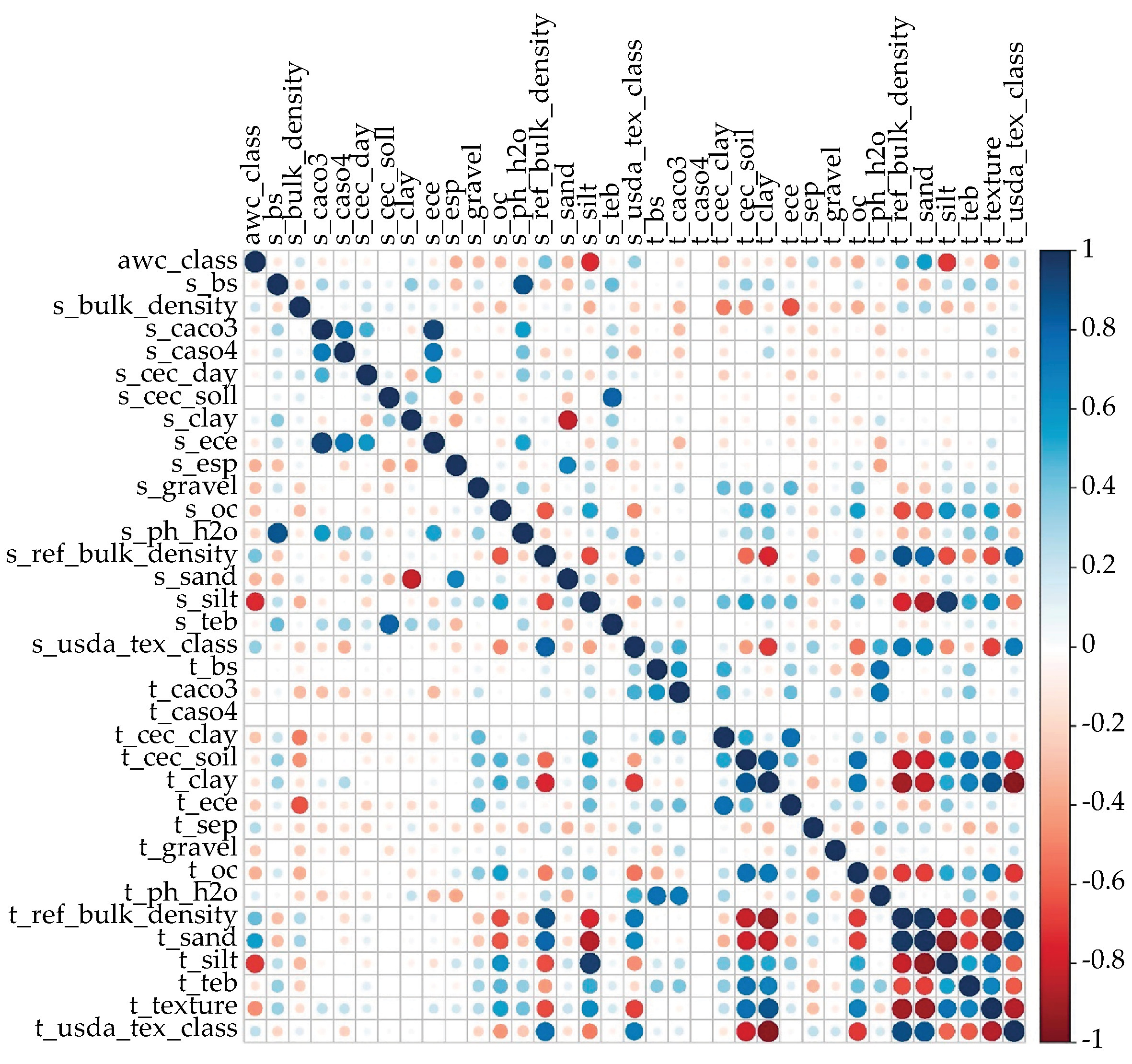

Supplement: S2 Fig — Collinearity matrix of candidate R. sibiricus predictor soil variables. Collinearity threshold is R > 0.75. Collinearity of variables increases with the depth of blue and red colors. Correlation strength increases with circle size. (TIF) [file pone.0297126.s002.tif]

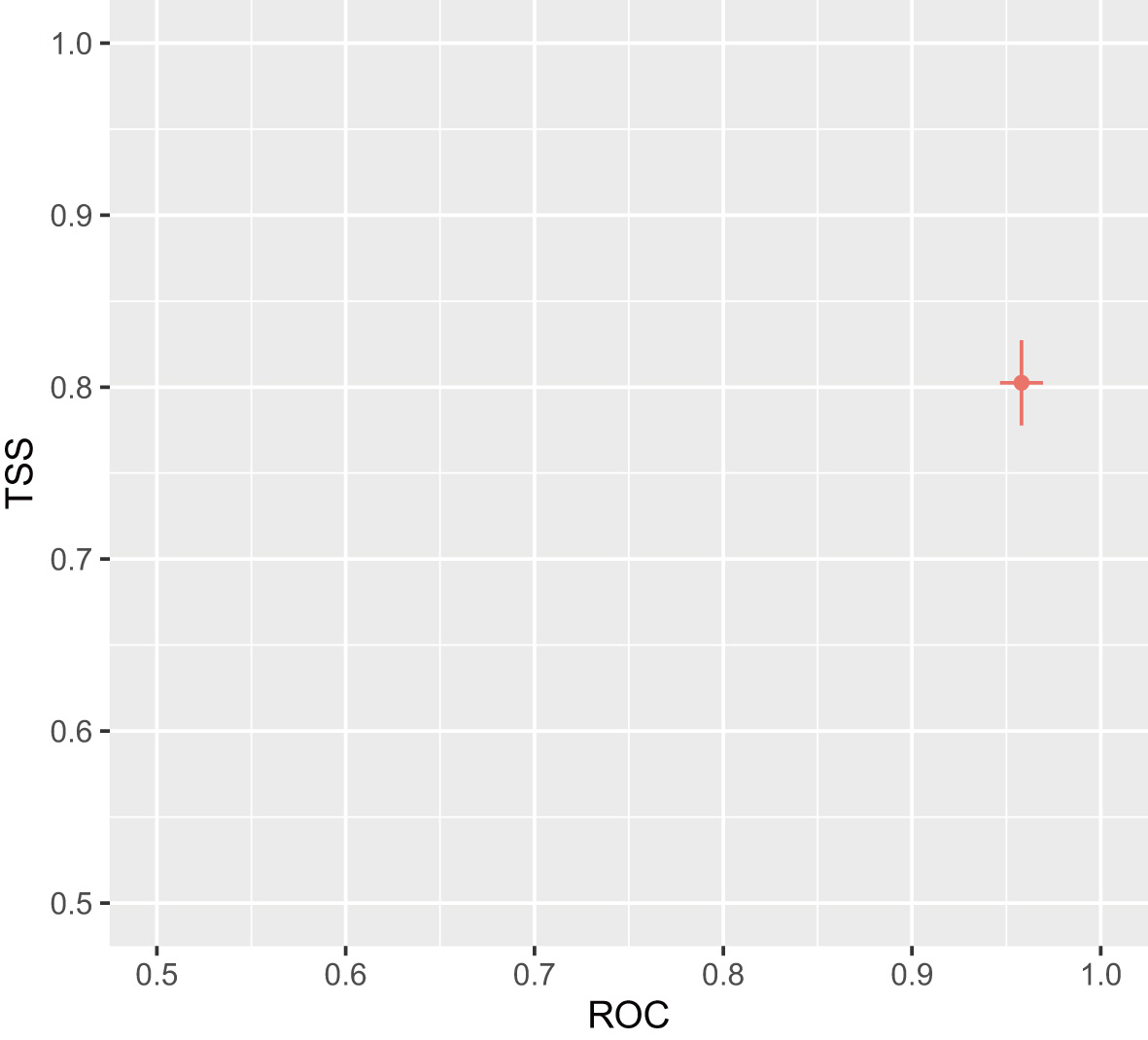

Supplement: S3 Fig — (TIF) [file pone.0297126.s003.tif]
